# Case of Intestinal Ohstruction or Occlusion, Lasting Thirty-Nine Days

**Published:** 1881-01

**Authors:** W. H. Lambart


### CASE OF INTESTINAL OBSTRUCTION OR OCCLUSION, LASTING THIRTY-NINE DAYS; TREATMENT BY SUBCUTANEOUS INJECTIONS OF MORPHIA; RECOVERY.

BY W. H. LAMBART, M. D., T. C. D., ETC.

On Monday, Sept. 15th, 1879, I was called to visit Mr. R. O——, aged sixty-four years. For months past, perhaps a year, he had no regular or healthy action of the bowels, but generally went to the closet four times daily, when a small liquid motion commingled with blood was passed. The appearance of the blood did not alarm him, as he had been treated by his club doctor on several previous occasions for bleeding piles.

The immediate previous history was as follows:—On Sept. 11th he felt uncomfortable, the usual calls to the closet having ceased. He took two rhubarb pills on his own responsibility. No effect being produced, he went to a chemist on Sunday 14th, and got a pill and draught, which he took in the shop, and brought away two more draughts, which the chemist directed him to take if the bowels did not act. The draughts were duly taken without result, so he took an ounce of castor oil; this did not succeed. Feeling very ill, he applied again to the chemist, who advised him to send for a doctor.

On my arrival my patient was in bed; had little sleep during the past night; complained of pain in the bowels, at times very severe, especially when following loud rumbling, which occurred frequently; abdomen swelled and tympanitic; vomiting every ten minutes. Temperature 100.05°, pulse 110. Ordered beef-tea often in small quantities, and an injection of soap and water, small in quantity and to be given with great gentleness.

16th.—Everything rejected from the stomach; symptoms as before. Ordered another injection, which brought away several small scybala, after which he expressed himself as feeling much better; also ordered a draught with quarter of a grain of morphia, to procure sleep, and to relieve the pains, which still came frequently.

17th.—Still vomiting; abdominal distension increasing. Saw in consultation my friend, Mr. Owen Thomas, who advised subcutaneous injections of morphia, and the foot of the bed to be raised six or eight inches. Injected quarter of a grain of morphia.

18th.—Much better night; less pain, less vomiting. Quarter of a grain of morphia in the morning, and again at night. To continue beef-tea, rice-water and arrowroot.

19th.—Feels comfortable; vomiting less frequent; if stercoraceous, without smell. Morphia, quarter of a grain morning and evening.

21st.—Pulse 115, temperature 98.2°. Morphia, one-third of a grain. Was sent for before my regular time for evening visit, as the patient was much worse and his friends thought he was dying. Morphia, one-third of a grain.

22d.—Passed in night some flatus; felt much better; pains nearly gone; vomited only three times during the day. Morphia, one-third of a grain morning and evening.

24th.—Pulse 120, temperature 97.5°. Has not vomited since the 22nd; more flatus passed; abdominal distension gradually increasing.

25th.—11 A. M.: Pains severe since four o'clock this morning. Pulse 128, temperature 98°. Distension still increasing. Injected half a grain of morphia. Sent for at 8 P. M. Much pain since four o'clock this afternoon. Pulse 148. Signs of collapse; still greater distension. Morphia, half a grain.

26th.—7.30 A. M.: Pulse 136. Not much pain in night, but turned suddenly in bed this morning, which caused much pain in left side. Feels weak; depressed. Ordered a little brandy and water; morphia half a grain. Distension still increasing. Visited at noon with my friend, Mr. Thomas. Very easy since morning. As abdomen was very distended we decided to let out the flatus. Abdomen assumed natural size. Morphia half a grain. Visited at 9 P. M.: Pulse 120; abdomen again filling rapidly. Morphia half a grain.

27th.—Pulse 124; temperature 98.9°. Passed good night; no sickness; no flatus. Distension still rapidly increasing. Morphia half a grain; repeated at 3 P. M. and at 10 P. M.

28th.—8 A. M.: Comfortable night. Pulse 124, with temperature 99°. No sickness, vomiting, or pain. Again tapped abdomen till about half its natural size. Morphia half a grain.—2.30 P. M.: Pulse 112, morphia half a grain.—9.30 P. M.: Pulse 110; morphia half a grain.

29th.—No sickness or pain. Morphia half a grain, morning, afternoon, and evening.

30th.—Much flatus in night and through the day. Morphia two-thirds of a grain three times.

Oct. 1st.—More flatus. Morphia two-thirds of a grain three times.

Oct. 2nd.—Pulse 120; temperature 100.2°. Much more flatus. A good deal of rumbling. The abdominal distension not increasing.

Between this date and Oct. 9th pulse varied from 120 to 106. Flatus passed occasionally. A little pain at times complained of. Thought on the 8th he could not recover. Given regularly three times every day two-thirds of a grain of morphia.

10th.—Felt sick. Had much pain in left side when he turned in bed. Morphia as before.

12th.—Felt like a large hard lump in left side, just above Poupart's ligament.

13th.—Abdomen increasing again. Pulse 120; temperature 98°. Much pain since 5 A. M. One grain of morphia three times a day.

14th, 15th, 16th, 17th, 18th.—Nothing particular to relate. One grain of morphia three times a day. Sometimes a little flatus, sometimes a very small quantity of black liquid fæces.

19th.—Two chambers nearly full of fæces, very liquid and black, passed. Such was the report I made to-day.

21st.—More fæces. Morphia three times a day till Nov. 5th, gradually reducing the dose; from that day till the 24th, twice a day, gradually reducing the quantity. As he complained of sickness of stomach and had bilious vomit, I gave solution of hydrochlorate of ammonia and extract of taraxacum on and after Nov. 7th with much advantage. Temperature as a rule, normal every day; pulse varying from 120 to 100.

Dec. 4th.—The dark color at length disappearing from the fæces, now becoming more natural in appearance.

Dec. 3d.—Only visit once daily and give morphia once. Motion nearly every day. Beginning to eat; everything he takes in small quantities.

25th.—Had roast goose and potatoes for dinner.

Jan. 4th, 1880.—No occasion to visit.

I was apprehensive of malignant tumour in this case; the history, the cachexy, the hard lump felt on the 12th Oct., all induced my thoughts to converge in this direction. The scybala removed by the injection on Tuesday, Sept. 16, were, I believe, in the rectum below the occlusion, and the small quantities of black fluid on the 17th and 18th of Oct. were too insignificant to allow me to suppose the obstruction was removed. The copious discharge on the 19th was evidence unmistakable. To gain space I have contracted the above notes as much as I could from a full record kept regularly every day.—*London Lancet*, Sept. 1880.

